# Literacy or Useless Knowledge? Associations Between Health Literacy and Lifestyle Among Adolescents

**DOI:** 10.3390/children12080978

**Published:** 2025-07-25

**Authors:** Bernadett Varga, Gábor Pál Stromájer, Dóra Heizler, Melinda Csima, Tímea Stromájer-Rácz

**Affiliations:** 1 Faculty of Health Sciences, Institute of Basics of Health Sciences, Midwifery and Health Visiting, University of Pécs, H-7621 Pécs, Hungary; bernadett.varga@etk.pte.hu (B.V.); dori.heizler@gmail.com (D.H.); 2 Institute of Education, Hungarian University of Agriculture and Life Sciences, H-7400 Kaposvár, Hungary; petone.csima.melinda@uni-mate.hu; 3 Institute of Diagnostic, Faculty of Health Sciences, University of Pécs, H-7621 Pécs, Hungary; timea.stromajer-racz@etk.pte.hu

**Keywords:** health literacy, health behavior, adolescents, health comprehension

## Abstract

**Background/Objectives:** Health literacy plays a fundamental role in adolescents’ health-related decisions and behaviors. The aim of our study was to assess the level of health literacy among 16–17-year-old students in Southern Hungary and to examine the associations between sociodemographic characteristics and health behaviors. **Methods:** This cross-sectional quantitative study was conducted in the autumn of 2024 in Baranya and Somogy counties. A total of 133 students completed a self-administered questionnaire including sociodemographic variables and health behaviors. Health literacy was measured using the validated HELMA-H instrument. Statistical analysis included chi-square tests, *t*-tests, and ANOVA (*p* < 0.05). **Results:** Overall, 62.7% of the students demonstrated adequate, while 37.3% demonstrated inadequate levels of health literacy. No significant association was found between overall health literacy and sociodemographic variables; however, partial associations were observed on specific subscales. Boys reported better access to health information (*p* = 0.037), while children of mothers with higher educational attainment scored better in comprehension (*p* = 0.042) and appraisal (*p* = 0.036). In the case of the numeracy subscale, children of mothers with the lowest educational level showed significantly better results (*p* = 0.006). Students with higher health literacy levels were less likely to smoke or consume caffeine; however, a reverse trend was observed regarding alcohol consumption. Physical activity showed a positive association with healthier behaviors (*p* < 0.05). **Discussion:** The use of digital technologies, interactive learning strategies, and the involvement of family members—especially mothers—may support the development of health-conscious decision-making in adolescents. Consequently, health education programs should focus not only on knowledge transfer but also on fostering critical thinking and decision-making skills.

## 1. Introduction

In recent years, Hungarian legislation has taken significant steps to protect the health of young people. In April 2025, the National Assembly passed a law prohibiting the sale and purchase of energy drinks to individuals under the age of 18 [1].

The purpose of this legislation is to safeguard the health of minors, given that the excessive caffeine and sugar content of energy drinks may have harmful effects on the developing body. An energy drink is defined as any beverage containing more than 15 mg of caffeine per 100 mL. According to a nationwide survey, 20% of 10–14-year-olds regularly consume such drinks, despite the fact that, according to the European Food Safety Authority, the available data are insufficient to determine a safe level of caffeine intake for children and adolescents [2,3].

The legal measures aimed at protecting the health of minors also include regulations concerning alcoholic beverages, which prohibit the sale or serving of alcoholic drinks to individuals under the age of 18. In entertainment venues and shops, refusing service is mandatory if there is any suspicion that the individual is underage [1].

In March 2025, the Hungarian government officially declared a zero-tolerance policy regarding the use and distribution of illicit drugs. The use and possession of illegal substances are strictly prohibited in schools and public educational institutions. The aim of these legal regulations is to prevent the development of substance use disorders, particularly among the adolescent population [4].

These regulations are not merely prohibitive in nature; they also convey an important societal message: the physical and mental well-being of adolescents is a matter of public interest. However, a key question remains—do young people possess the skills and knowledge necessary to recognize the health risks underlying these regulations and to make informed decisions accordingly? The capability required for this is provided by health literacy.

Health literacy is a complex, multidimensional concept that plays a fundamental role in health promotion and disease prevention. According to the World Health Organization (WHO), health literacy encompasses the cognitive and social skills that determine the motivation and ability of individuals to gain access to, understand, and use health information in ways that promote and maintain good health [5].

Adolescents represent an especially vulnerable group, as this life stage is marked by significant biological, psychological, and social changes. During this developmental period, the ability to understand and apply health information is of critical importance, as it influences both current and future health-related behaviors [6,7].

Low levels of health literacy are associated with adverse health outcomes, such as poor decision-making, engagement in health-risk behaviors (e.g., smoking, alcohol consumption), and limited access to preventive health services [8,9].

In Hungary, Horváth et al. (2021) [10] validated the Health Literacy for Adolescents-Hungarian version (HELMA-H) questionnaire, which serves as a valuable tool for assessing adolescents’ health literacy. Although the HELMA-H questionnaire was validated relatively recently in Hungary, it has only been applied in a few empirical studies to date. The present research represents one of the first applications of the HELMA-H in the adolescent population of Southern Hungary, thereby not only exploring the level of health literacy but also contributing to the further validation and usability of the instrument in the Hungarian context [10].

When implementing health literacy interventions in school settings, it is essential to consider the principles of health equity. Naccarella and Guo (2022) emphasize that the planning of such programs should address not only curriculum content, but also cultural specificities, student–teacher interactions, and the broader social context [11]. These considerations are crucial to ensuring that health literacy initiatives are truly accessible and effective for all students, and do not inadvertently exacerbate existing health disparities.

Consistent with this, other studies also confirm that school-based programs can effectively enhance adolescents’ willingness to seek help and foster the development of health-conscious behaviors [12].

The primary setting for a child’s upbringing is the family, which plays a pivotal role in the process of socialization. It is within the family environment that children acquire their first relational patterns, communication skills, and behavioral norms—including those related to a health-conscious lifestyle. The family shapes their identity, emotional security, and the understanding of social relationships and roles. It is also where children learn self-regulation, conflict resolution, and various forms of communication, all of which have a lasting impact on their cognitive and behavioral development [13,14].

The integration of family-acquired values into daily life facilitates successful adaptation to subsequent socialization contexts and adherence to social norms. As children enter preschool, the family’s influence gradually diminishes, giving way to the growing impact of peers and educators. Nonetheless, health education and the promotion of healthy lifestyles must remain continuous across all developmental and institutional contexts [15].

Today, one of the key factors in this process is the appropriate use of digital devices. The intensive or problematic media usage behavior of parents and educators can serve as a model for children’s own media habits [16]. Digital media has become an integral part of family life [17].

However, navigating the digital space poses significant challenges. According to a 2023 study, members of Generation Z often use the internet to search for health-related information, yet they demonstrate lower levels of health autonomy and reduced willingness to actively seek out such information—highlighting the risks of digital misinformation [18,19]. Moreover, media use and digital competencies play a crucial role in the prevention of non-communicable diseases as well [20].

Integrating interactive learning methods into health education represents a promising approach to enhancing health literacy. In their comprehensive review, Mancone et al. (2024) emphasize the benefits of using digital technologies—such as mobile applications and virtual reality—for expanding adolescents’ health knowledge [21].

Parental health literacy also exerts a significant influence on children’s health behavior. A 2024 Hungarian study demonstrated that higher parental health literacy and educational attainment positively impact the development of health-preserving habits in preschool-aged children [22,23,24]. This finding holds particular significance, as numerous studies—including Felitti’s classic ACE study (2002) [25] and the comprehensive, representative U.S. research conducted by Swedo et al. (2023) [26]—have clearly shown that childhood health status and adverse experiences substantially affect physical and mental health in adulthood. This relationship not only shapes individual quality of life but also serves as a critical factor from both national economic and public health policy perspectives [25,26,27].

International research increasingly highlights that adolescent health literacy is influenced by a wide range of factors. A comprehensive analysis has shown that sociodemographic variables such as gender, age, and socioeconomic background significantly affect the understanding and application of health-related information [7,28]. A cross-sectional study conducted in China reported similar findings, indicating that the level of digital health literacy is significantly influenced by age, educational attainment, and online information-seeking behaviors [29].

A strong association would be assumed between physical activity and health literacy, but this is not consistently confirmed in all cases. Therefore, it is advisable to develop the various dimensions of health literacy separately. Moreover, the school environment and its associated programs play a crucial role in fostering health literacy, particularly among disadvantaged students [30]. In addition, the influence of parents and community-based interventions significantly impacts young people’s health behaviors [31].

Engaging children in the research process and in interactive interventions—such as those implemented through the LifeLab program—can support the development of sustainable and contextually relevant health education strategies [32].

Based on the recognition of health literacy as a social determinant of health, the aim of this study is to explore the health literacy levels of 16–17-year-old students in Southern Hungary, as well as its associations with sociodemographic characteristics and health behaviors. Our findings may contribute to the development of targeted interventions and educational programs that support the improvement of adolescents’ health awareness, thereby enhancing the effectiveness of legislative regulations.

## 2. Materials and Methods

The research employed a quantitative, descriptive, cross-sectional design, aiming to explore the associations between adolescents’ health literacy, health behaviors, and sociodemographic characteristics. The study was conducted between September and November 2024 in the Hungarian counties of Baranya and Somogy.

A total of 133 secondary school students aged 16–17 participated in the study, selected through convenience sampling from grammar schools, vocational secondary schools, and trade schools. Inclusion criteria included active student status, age between 16 and 17 years, and provision of informed consent. Students with chronic illnesses or those unable to complete the questionnaire independently were excluded from the sample.

Data collection was conducted using a questionnaire package that included both self-developed questions and a validated instrument. The custom-designed questionnaire comprised sections on sociodemographic variables (gender, age, place of residence, parents’ educational attainment, school type, and financial situation) as well as on health behavior (physical activity, alcohol consumption, smoking habits, and caffeine consumption).

The validated HELMA-H questionnaire was employed in the study, which was adapted and validated for the Hungarian adolescent population by Horváth et al. (2021) [10]. Given the limited empirical application of this instrument in Hungary to date, our study is among the first to utilize this tool to assess the health literacy level of 16–17-year-old students. The original HELMA questionnaire was developed by Ghanbari et al. in 2016 [10,33].

### Description of the Applied Questionnaire

In this study, we used the HELMA-H (Health Literacy for Adolescents-Hungarian version) questionnaire to assess health literacy. This instrument was specifically developed and adapted for evaluating adolescent health literacy within the Hungarian context. Its purpose is to provide a comprehensive overview of the extent to which students are able to access health-related information, understand it, and apply it in practice.

In addition, the tool evaluates communication skills, as well as numeracy and decision-making abilities, all within a health-related context.

The questionnaire consists of eight distinct subscales: self-efficacy, access, reading, understanding, appraisal, use, communication, and numeracy. The first 41 items are rated on a five-point Likert scale, where respondents indicate the extent to which specific statements apply to them. Response options range from “never” to “always,” with scores from 1 to 5 points.

The final three items of the questionnaire are open-ended numerical tasks, in which a correct answer is awarded 5 points, while an incorrect response receives 1 point. The questions associated with each subscale, as well as their respective scoring ranges, are detailed in Table 1.

Based on the questionnaire results, each individual’s health literacy level—referred to as the HELMA-H score—can be determined using the following formula:

$$Score=\frac{Raw\;score-Minimum\;possible\;score}{Maximum\;possible\;raw\;score-Minimum\;possible\;raw\;score}\;\times \;100$$

After summing the questionnaire scores, respondents’ health literacy levels were categorized into four groups based on score thresholds commonly used in the literature:

- Excellent: 84.01–100 points;
- Adequate: 66.01–84 points;
- Problematic: 50.01–66 points;
- Inadequate: 0–50 points.

The first two categories—“excellent” and “adequate”—are collectively interpreted as desirable health literacy levels, whereas those falling into the “problematic” and “inadequate” categories are considered to have limited health literacy. In the latter cases, individuals are less capable of interpreting and applying health-related information, which may hinder informed and health-conscious decision-making.

The internal reliability of the instrument was assessed using the Cronbach’s alpha method [34]. The overall Cronbach’s alpha for the entire questionnaire was 0.888, indicating excellent internal consistency. Most of the individual scales exceeded the accepted threshold of 0.70, which is considered the minimum acceptable level for psychometric instruments. Exceptions included the self-efficacy scale (α = 0.612) and the numeracy scale (α = 0.554), which demonstrated lower reliability levels. It is important to note that the Cronbach’s alpha value reported for the numeracy subscale (α = 0.554) is only of limited interpretability. The three items included in this subscale measure different cognitive abilities—such as calculation skills, percentage interpretation, and graph reading—which are not necessarily expected to correlate with each other. Therefore, the application of Cronbach’s alpha in this context is not methodologically fully appropriate. For assessing the reliability of numerical skills, item-level analyses or test–retest reliability may be more suitable approaches. Nevertheless, these scores may still be considered acceptable for preliminary assessments or educational research purposes [35] (Table 2).

In summary, the HELMA-H questionnaire proved to be a suitable tool for the multidimensional assessment of adolescent health literacy, especially in school settings where evaluating students’ awareness and skills is critical for effective health promotion and prevention efforts [10].

## 3. Results

A total of 133 secondary school students aged 16–17 from educational institutions in Baranya and Somogy counties participated in the study. Based on the gender distribution of the sample, 64.7% of the participants were female, while 35.3% were male. Regarding the type of school, 57.1% of the students attended grammar school, 26.3% were enrolled in technical or vocational secondary schools, and 16.5% studied in vocational training schools. In terms of the place of residence, 52.6% lived in cities, while 47.4% resided in towns or villages.

Concerning the mothers’ educational attainment, 12% of the respondents reported that their mothers had only primary education, 61% had secondary education, and 27% had higher education qualifications. In the case of fathers, secondary education was the most common (more than 60%), while 33.8% had higher education degrees, and those with only primary education were represented in the lowest proportion (4.5%).

The level of health literacy was measured using the validated HELMA-H questionnaire. The scores ranged from 31 to 92, with an average total score of 67.31 (SD = 10.918), which is considered an adequate level on the instrument’s scale. Based on the obtained results, students were categorized into four groups: only 3.8% achieved an “excellent” level of health literacy, 58.6% reached an “adequate” level, 29.3% were classified as having a “problematic” level, while 8.3% showed an “inadequate” level of health literacy.

Following the recommendations in the literature, we formed two broader categories based on these four: the desirable health literacy group (those in the “adequate” or “excellent” categories), which included 62.4% of the adolescents, and the limited health literacy group (those in the “problematic” or “inadequate” categories), comprising 37.6% of the participants (Table 3).

We compared the adolescents’ total health literacy scores with various sociodemographic characteristics. We found only minimal differences between genders, with girls scoring slightly higher than boys. In terms of age, the results of 16- and 17-year-olds were similar.

Regarding the type of residence, students living in small towns and cities achieved the highest scores, while those living in rural areas had slightly lower results. Based on parents’ educational attainment, children of parents with higher levels of education performed better.

The family’s financial situation also influenced the average scores, with students from lower-income families scoring lower than those living in average or above-average conditions. However, this was also affected by the parents’ highest level of education. Based on school type, grammar school students performed the best, while students attending vocational secondary schools had the lowest scores.

However, our statistical analyses did not reveal any significant associations between the health literacy scores and any of the examined sociodemographic variables (*p* > 0.05).

When examining the variables based on the HELMA categories, higher health literacy was observed among girls, 17-year-olds, students living in urban areas, grammar school students, children of parents with higher educational attainment, and students from above-average financial backgrounds. However, these differences were not statistically significant (*p* > 0.05) (Table 4).

The HELMA-H questionnaire is a multidimensional measurement tool; therefore, we also analyzed the results according to its subscales. We found varying average scores across the eight subscales of the HELMA-H questionnaire. The highest value was measured on the “Understanding” scale, while the “Numeracy” scale yielded the lowest score (Table 5).

Among the sociodemographic variables, we found statistically significant differences in relation to gender and the mother’s level of education (*p* < 0.05). On the “Access” scale, boys scored significantly higher (M = 20.21; SD = 3.103) than girls (M = 18.94; SD = 3.866; t = −2.110; *p* = 0.037).

The mother’s educational attainment had a significant effect on three subscales: understanding (F = 3.256; *p* = 0.042), appraisal (F = 3.419; *p* = 0.036), and numeracy (F = 3.863; *p* = 0.023). On the understanding scale, a gradual increase was observed corresponding to the level of education: children of mothers with higher education achieved the highest scores (M = 42.03; SD = 4.414), while children of mothers with vocational training obtained the lowest scores (M = 38.75; SD = 6.801). A statistically significant difference was found between the vocational and high school graduate groups (*p* = 0.036), as well as between the vocational and higher education groups (*p* = 0.018).

On the appraisal scale, children of mothers with a high school diploma achieved the highest scores (M = 19.00; SD = 2.780), while the lowest scores were recorded among children of mothers with vocational education (M = 17.19; SD = 4.314; *p* = 0.011).

Interestingly, on the numeracy scale, children of mothers with the lowest educational attainment performed the best (M = 12.13; SD = 4.218), whereas those whose mothers had completed secondary education achieved the lowest average score (M = 9.75; SD = 3.890; *p* = 0.006).

In our study, we also inquired about health-related behaviors. A total of 65.4% of participants reported engaging in physical activity at least three times per week, while 34.6% exercised only occasionally or not at all. Regular physical activity had a positive impact on risk behaviors: students who exercised regularly smoked significantly less (*p* = 0.018) and also reported lower levels of alcohol (*p* = 0.023) and caffeine consumption (*p* = 0.033).

Regarding smoking, 21.1% of students stated that they smoked at least weekly, while the rest smoked only occasionally or not at all. Among the sociodemographic variables, maternal educational attainment showed a significant association with smoking habits (*p* = 0.017). The highest rate of smoking (46.9%) was observed among students whose mothers had completed vocational education, while only 14.7% of those whose mothers had a higher education degree reported regular smoking.

With respect to alcohol consumption, 32.3% of respondents reported drinking alcohol weekly or more frequently. In terms of caffeine intake, 48.9% of students stated that they consumed caffeine-containing beverages on a daily basis.

We also found associations between health literacy levels and health behavior patterns: adolescents with higher levels of health literacy smoked less frequently (*p* = 0.020) and consumed less caffeine (*p* = 0.007). Surprisingly, in the case of alcohol consumption, students with better health literacy reported a more frequent intake of alcoholic beverages (t = 2.042; *p* = 0.043) (Table 6).

Sociodemographic factors also influenced health behavior patterns. The frequency of physical activity was affected by the place of residence (*p* = 0.040), school type (*p* = 0.029), and financial status (*p* = 0.025). Students attending grammar schools, those living in better financial conditions, and those residing in larger cities or county seats engaged in a more active lifestyle.

## 4. Discussion

The aim of the study was to assess the health literacy of 16–17-year-old secondary school students in Southern Hungary, with a particular focus on the impact of sociodemographic characteristics and health behavior patterns. Based on the results of the HELMA-H questionnaire, 62.7% of the students demonstrated an adequate level of health literacy, while 37.6% were found to have an inadequate level.

This ratio is considered moderate in comparison to international results. In a representative study conducted in Germany among 15–19-year-olds, the proportion of adolescents with inadequate health literacy was 47%, with young people from lower social strata being particularly at risk [36]. Similar findings were reported in Austria, where 40–45% of adolescents were found to have low health literacy levels, especially among those attending vocational training institutions [9]. In contrast, in Scandinavian countries—such as Norway—the level of health literacy among young people was higher, with more than 70% of students achieving adequate health literacy [37].

The results from the Hungarian sample thus indicate that, although the level of health literacy is not exceptionally low in comparison to international data, a significant proportion (more than one-third) of students still lack sufficient knowledge and skills to make informed health-related decisions. This finding underscores the need for health education programs and school-based interventions, particularly with the aim of reducing social inequalities.

### 4.1. The Impact of Sociodemographic Factors

In the present study, no statistically significant associations were found between sociodemographic variables and overall health literacy. However, trends indicated that girls, 17-year-old students, those living in urban areas, grammar school students, and children of parents with higher educational attainment generally achieved higher health literacy scores. Notably, maternal educational attainment emerged as an influential factor across several subscales, suggesting that the HELMA-H questionnaire is capable of sensitively reflecting certain sociodemographic influences.

This correlation aligns with previous international research findings. For instance, a study conducted by Loer et al. in Germany identified a strong relationship between maternal educational attainment and health literacy: children of parents with higher levels of education scored significantly higher on health literacy scales [36]. Similar results were reported in a cross-sectional study from China, which demonstrated a significant association between parental education and adolescents’ digital health literacy [29]. Findings from Scandinavian countries [37] also suggest that the quality of the school environment—including teacher competence and school infrastructure—meaningfully contributes to the development of students’ health literacy.

Similar trends can be observed in Hungary. According to a study by Csima et al., students’ levels of health literacy are primarily shaped by family background—especially maternal education—and the school environment [22]. These findings suggest that social inequalities already exert a significant influence on health-related knowledge and behavior during adolescence, potentially determining health outcomes in the long term.

Gender differences were observed specifically in the “access” subscale, where boys scored significantly higher (*p* = 0.037). This somewhat contradicts earlier international studies: for instance, Manganello and You & Ahn found that girls generally exhibit higher levels of health literacy, particularly in terms of communication and interpretive skills [6,7]. A possible explanation for the current finding is that the concept of “access” primarily refers to the ability to locate and use digital sources. In this domain, boys often demonstrate greater confidence and practicality—especially regarding the technical aspects of information retrieval.

This is supported by a recent German study, which found that while girls tend to be more empathetic and reflective when engaging with health-related content, boys navigate digital platforms more effectively and rely on a wider range of sources [36]. A study conducted in Taiwan also confirmed that boys’ technological self-confidence is more strongly correlated with certain dimensions of digital health literacy, particularly the “access” and “application” subscales [38]. At the same time, girls generally demonstrate a closer association with social and emotional aspects, such as willingness to seek help or adopting preventive health attitudes [37].

This result therefore does not necessarily contradict the previous literature; rather, it suggests that the various dimensions of health literacy (such as access, understanding, application, and decision-making) may develop differently across genders. To interpret gender differences accurately, it is advisable to employ complex, multidimensional models of health literacy.

In addition to gender, another sociodemographic factor—the mother’s educational attainment—also yielded unexpected results, particularly in the case of the numeracy subscale. Interestingly, children of mothers with the lowest level of education achieved the highest scores on this subscale. At first glance, this seems to contradict previous research, which has found that higher parental education—especially maternal—has a positive influence on children’s health literacy [22,28]. A possible explanation is that these students may have acquired numerical skills through practical life experiences, such as early assumption of responsibilities or vocational training-related education. It is also important to consider that the numeracy subscale includes various types of tasks (calculation, percentage interpretation, graph reading), which may be influenced by different cognitive or social background factors. Therefore, a high score on this subscale does not necessarily indicate a generally higher level of health literacy but rather reflects the specific characteristics of the subscale and the students’ experiences outside formal education.

### 4.2. Health Behavior Patterns and Health Literacy

In relation to health behavior, a significant association was found between physical activity and self-efficacy (F = 5.651; *p* = 0.004), indicating that regular exercise positively affects students’ health literacy, particularly in the area of self-efficacy. Caffeine consumption and other lifestyle habits also correlated with various health literacy subscales. Students involved in regular sports achieved higher self-efficacy scores, suggesting that physical activity benefits not only physical well-being but also cognitive and psychosocial aspects of health-related decision-making. This aligns with the conclusions of Kesić et al. and Mancone et al., who emphasized that exercise strengthens health literacy components essential for managing health behaviors [21,30]. A Finnish longitudinal study similarly found that adolescents engaging in weekly activity are more likely to have higher health literacy and stronger internal control [37]. Additionally, a Canadian study showed that an active lifestyle supports the understanding and application of health information, as physically active youth are more receptive to preventive messages and more willing to adopt behavioral changes [39]. Thus, the positive effects of physical activity on health literacy are both direct and mediated through motivational and cognitive mechanisms.

We found a significant difference between smoking and the application subscale (t = 2.159; *p* = 0.033), with non-smoking students achieving higher scores. This result supports previous findings suggesting that low health literacy is often associated with health-damaging behaviors, such as smoking [8,9]. The application dimension—which refers to the practical use of health-related information—sensitively reflects behavioral differences: better application skills are more likely to be associated with avoiding smoking and making healthier decisions.

In contrast, an unexpected result emerged in the case of alcohol consumption: students who consumed alcohol scored higher on the application subscale (t = 2.042; *p* = 0.043), which may appear paradoxical at first glance. A similar phenomenon was reported by Demjén et al., who noted that certain health-risk behaviors—especially during adolescence—do not necessarily stem from a lack of information. Instead, psychosocial factors such as peer pressure, stress-coping strategies, and identity formation also play a significant role [40].

According to a 2023 Spanish study, alcohol consumption did not show a negative correlation with health literacy levels, as students were able to interpret health-related information, but their decisions were more strongly influenced by social and emotional factors [41]. Our results suggest that a higher application score does not necessarily translate into health-promoting behavior, especially when underlying factors such as academic pressure or peer expectations are present. Among students with highly educated parents—especially those attending grammar schools—alcohol use may function as a maladaptive coping strategy for dealing with academic or social pressures, despite their high level of health knowledge and information-processing skills.

This highlights the fact that certain dimensions of health literacy (e.g., application) do not always directly reflect the quality of health behavior. This observation aligns with the model proposed by Paasche-Orlow and Wolf, which suggests that psychological, social, and environmental mediators intervene between knowledge and behavior, potentially weakening the direct effect of literacy on actual practices [42].

Regarding caffeine consumption, no significant difference was observed in HELMA-H scores; however, a significant difference was found between the HELMA categories (adequate vs. inadequate health literacy) (*p* = 0.007). Among students who did not consume caffeine, a lower proportion showed inadequate health literacy, suggesting that adolescents with higher levels of health literacy tend to make more conscious lifestyle decisions.

This finding is consistent with results from other international studies, which indicate that an excessive consumption of caffeinated beverages—especially in the form of energy drinks—is often associated with underestimation of health risks and lower levels of health literacy. An Italian study found that among high school students who regularly consumed energy drinks, the ability to recognize health risks was lower, particularly in relation to caffeine overdose, sleep disturbances, and anxiety [43].

Similar results were reported in a Canadian study, where the level of caffeine consumption was inversely related to the comprehension and critical evaluation of health-related information [44]. The researchers emphasized that a high caffeine intake among adolescents is often part of a broader pattern of health-risk behavior, frequently occurring alongside smoking and alcohol consumption.

It is worth noting that, according to the European Food Safety Authority, there is insufficient scientific evidence to establish a safe level of caffeine intake for children and adolescents. Therefore, emphasizing the importance of moderation should be a key focus in preventive health programs [45]. The present findings thus suggest that higher health literacy involves not only the possession of information but also its practical application in everyday lifestyle decisions.

### 4.3. Sociodemographic Factors and Health Behavior

The frequency of physical activity was influenced by type of residence (*p* = 0.04), school type (*p* = 0.029), and family financial status (*p* = 0.025), highlighting that certain sociodemographic factors significantly shape adolescents’ activity levels. Students from above-average financial backgrounds engaged in sports more often, while the highest inactivity rates were among vocational school attendees. Interestingly, rural students were more likely to engage in daily activity, whereas urban youth typically reported 4–5 h per week. International studies report similar patterns: a European analysis found physical activity closely tied to socioeconomic status and school or community support [46]. Urban adolescents often lack space and time for exercise, while rural youth benefit from natural environments and alternative transport modes that encourage movement [47]. School type also plays a role: vocational students often receive less formal health education and have fewer structured activity options. This shows that activity is shaped not only by individual choice but also by institutional and infrastructural conditions [48]. Smoking habits were significantly linked to school type and maternal education: grammar school students and those with highly educated mothers showed lower smoking rates. This aligns with European, Chinese, and South Korean findings that parental education strongly influences adolescent health behavior through role modeling and communication [22,27,49]. The mother’s role appears especially influential, as her impact on daily routines and health decisions has a strong effect on adolescents’ health literacy, underscoring her central role in shaping children’s health understanding and behaviors [50].

### 4.4. Connections Between Physical Activity and Other Behaviors

Among students engaging in at least 4–5 h of physical activity per week, 84% reported being non-smokers, whereas the proportion of smokers was significantly higher among inactive peers. A similar trend was seen for alcohol consumption (*p* = 0.023) and caffeine intake (*p* = 0.033): the most active students were most likely to abstain from both. These findings suggest a positive link between regular exercise and favorable health behaviors. This correlation is well documented internationally, showing that adolescents involved in sports are less likely to engage in harmful behaviors. An HBSC analysis across 36 countries found that physical activity frequency is negatively associated with smoking and alcohol use, especially among those participating in moderate to vigorous exercise [51]. Additionally, WHO global data (2018) confirm that active adolescents are more inclined toward health-conscious habits. This is partly because sports often occur in social settings that encourage norm-conforming and preventive behavior. Moreover, the psychological benefits of exercise—such as better stress management, enhanced self-control, and increased endorphins—can offer healthier alternatives to risky behaviors [52]. It has also been noted that physically active adolescents tend to be more critical of energy drinks and other caffeine products. A New Zealand study found lower energy drink consumption among active youth, attributed to their higher health literacy and stronger community norms [53].

### 4.5. Limitations and Future Directions

One of the strengths of our study is the use of a validated questionnaire with detailed subscales (HELMA-H), which is still relatively uncommon in Hungarian-language applications. This study may thus contribute to the expansion of domestic assessment tools and allow for international comparisons, especially in regions where targeted measurement of adolescent health literacy is lacking. Furthermore, adolescents represent a particularly important target group for the development of health awareness and the foundation of future health behaviors [54].

Among the limitations of our study is the small sample size (n = 133), which restricts the generalizability of the findings and limits the strength of statistical inferences. This is especially relevant, as sociodemographic differences could be explored in greater depth with a larger sample [55]. Additionally, due to the cross-sectional design of the study, causal relationships cannot be established—only associations can be identified.

One limitation of our study is that we primarily applied bivariate statistical methods, which did not allow us to disentangle the overlapping effects of background variables (e.g., school type, parental educational attainment, place of residence). This means that the associations observed with certain variables may be influenced by confounding factors, potentially distorting the interpretation of the results. It would be particularly important for future research to apply multivariable models (e.g., logistic regression), which would enable the control of confounding variables and allow for a more precise identification of independent effects.

In addition to quantitative data, the application of qualitative methods (such as interviews and focus groups) would also be necessary to gain a deeper understanding of the social and cultural contexts of health perceptions—especially among disadvantaged youth [56].

In our analysis, the continuous scores of the HELMA-H questionnaire were categorized into discrete groups. While this approach facilitates the comparability and interpretability of the results, it may have led to some loss of information and a reduction in statistical power. In future studies with larger sample sizes, it would be advisable to retain continuous variables and apply multivariable models to more accurately isolate the effects of background variables.

Furthermore, future research should place a stronger emphasis on the separate measurement of digital health literacy, given that a significant proportion of young people rely on online sources for health-related information. The use of tools such as the eHealth Literacy Scale (eHEALS) could complement the structural strengths of the HELMA-H tool and provide deeper insights into digital access and critical evaluation of online health information [57].

Overall, our research highlighted the strong interrelationship between health literacy and health behavior and showed that numerous sociodemographic factors influence these either directly or indirectly. The findings reinforce the necessity of targeted school-based and family-oriented health promotion programs, particularly for disadvantaged groups.

Such programs should not be limited to the mere transmission of knowledge, but must also aim to enhance self-efficacy, critical thinking, and the ability to evaluate health information sources—skills that fundamentally shape health-related decision-making in the long term.

## 5. Conclusions

The results of our study indicate that the overall health literacy level of secondary school students in Southern Hungary is adequate; however, certain groups—particularly those from lower socioeconomic backgrounds—require further support and development. Health behavior patterns, such as regular physical activity, abstinence from smoking, and reduced caffeine consumption, showed strong associations with specific domains of health literacy. These findings suggest that preventive programs should focus not only on knowledge dissemination but also on promoting positive lifestyle habits.

A particularly notable finding is the impact of maternal educational attainment, which emerged as a significant determinant across several subscales. This supports the notion that family background—especially maternal health awareness—plays a lasting role in shaping adolescents’ health behavior. In the future, it would be beneficial to design interventions that actively involve families in the health promotion process.

It is important to note that the findings of this study are consistent with the international literature, which emphasizes that health literacy is not merely an individual competence, but a social construct shaped by the school environment, media use, and family and social capital [54,56]. This highlights that school-based health education can only be truly effective if it is reinforced at both the level of educational policy and within local community practices. Targeted, multi-level interventions—simultaneously addressing students, their families, and educators—may be crucial in fostering health-conscious future generations.

The study also highlights the importance of focusing on the development of digital health literacy in future health education, as young people are increasingly turning to online platforms for information. The ability to interpret, critically evaluate, and apply health-related information forms the foundation not only for current but also for future public health challenges. In light of these considerations, our research may contribute to the evidence-based advancement of school health promotion—particularly in efforts to reduce social inequalities and enhance the effectiveness of early interventions.

Our practical recommendations are as follows:

- Strengthening school-based health promotion: Lessons, theme days, or interactive workshops focused on developing health awareness can effectively support students in interpreting and applying health-related information.
- Involving parents in prevention: In light of the research, it would be justified to organize programs that also reach parents—especially mothers—through community lectures or online training sessions. Naturally, educating parents is also of utmost importance.
- Developing digital awareness: Boys’ technological confidence may provide an advantage in accessing health information. This skill should be intentionally developed in all students, for example, through the introduction of media literacy classes.
- Local-level health promotion initiatives: In order to reduce regional disparities, it is essential for local governments, schools, and civil society organizations to work together on targeted, community-based health promotion programs.

In conclusion, the development of school-based health education, the involvement of families, and the conscious enhancement of digital competencies can all contribute to enabling young people to navigate the world of health information with greater confidence and responsibility. Our study may thus contribute to improving health literacy levels in Hungary and support the design of more targeted health policy interventions.

## Figures and Tables

**Table 1:** HELMA questionnaire items and scores by scale (Horváth et al., 2021) [10].

Scale	Number of Items	Point Range
Self-efficacy	4 (1–4)	4–20 points
Access	5 (5–9)	5–25 points
Reading	5 (10–14)	5–25 points
Understanding	10 (15–24)	10–50 points
Appraisal	5 (25–29)	5–25 points
Use	4 (30–33)	4–20 points
Communication	8 (34–41)	8–40 points
Numeracy	3 (42–44)	3–15 points
Sum	44 questions	44–220 points

**Table 2:** Cronbach’s alpha values for the HELMA-H questionnaire and its subscales.

Scale	Number of Items	Cronbach’s Alpha (n = 133) HELMA H
Self-efficacy	4	0.612
Access	5	0.759
Reading	5	0.702
Understanding	10	0.838
Appraisal	5	0.753
Use	4	0.711
Communication	8	0.731
Numeracy	3	0.554
Sum	44	0.888

**Table 3:** Creating of HELMA categories (n = 133).

HELMA Category	Points	Combined Category	Points
Excellent −3.80%			
	84.01–100		
		
Adequate −58.60%		Desirable	
	66.01–84	−62.40%	66.01–100
		
Problematic −29.30%			
	50.01–66		
Inadequate −8.3%	0–50	Limited −37.76%	0–66

**Table 4:** Average HELMA scores by sociodemographic distribution (n = 133).

		N	Mean	SD	Minimum	Maximum
Gender	Male	62	68.06	10.452	31	89
	Female	71	66.66	11.343	41	92
Age	16 years	56	67.36	10.698	37	89
	17 years	77	67.28	11.145	31	92
County	Baranya	76	68.03	10.608	31	89
	Somogy	57	66.36	11.343	41	92
Type of school	Vocational school	22	64.87	13.252	41	86
	Technical school	35	65.02	13.308	31	92
	Gymnasium	76	69.08	8.542	47	83
Financial status	Below average	65	63.89	10.202	31	89
	Average	63	67.64	11.516	37	92
	Above average	5	67.26	14.017	41	76
Mother’s education	≤Primary	8	70.9	7.732	58	81
	Vocational	24	65.23	14.796	31	89
	Secondary	44	65.42	10.678	37	92
	University	57	69.15	9.277	47	83
Father’s education	≤Primary	6	65.74	16.46	37	81
	Vocational	37	67.49	11.756	41	89
	Secondary	45	65.91	10.979	31	92
	University	45	68.78	9.415	47	80
Type of residence	Village	47	67.02	11.816	41	92
	Small town	22	67.8	9.814	49	82
	City	64	67.36	10.749	31	89

**Table 5:** Average scores of HELMA subscales (n = 133).

	N	Mean	Std. Deviation	Minimum	Maximum
Access	133	19.62	3.524	5	25
Reading	133	19.29	3.375	7	25
Understanding	133	40.86	5.638	24	50
Appraisal	133	18.47	3.316	9	25
Use	133	12.30	3.555	4	20
Communication	133	28.86	4.948	16	40
Self-efficacy	133	15.29	2.647	5	20
Numeracy	133	10.49	4.072	3	15

**Table 6:** Smoking, caffeine and alcohol using comparison to HELMA-H Category.

HELMA-H Category				
	Category	Desirable	Limited	*p*
Caffeine consumption	Never	24%	6%	
	Daily	17%	34%	0.07
	Weekly	59%	60%	
Smoking	Smoker	22%	42%	
	Non-smoker	77%	58%	0.02
		Mean/SD	t-value	
Alcohol consumption	Drinks alcohol	19.8 ± 3.433		
	Never drinks alcohol	18.62 ± 3.259	2.042	0.043

## Data Availability

The data of the research are available from B.V. and G.P.S.
